# Quantitative Proteomic Analysis of Gingival Crevicular Fluid in Different Periodontal Conditions

**DOI:** 10.1371/journal.pone.0075898

**Published:** 2013-10-01

**Authors:** Carina M. Silva-Boghossian, Ana Paula V. Colombo, Marcia Tanaka, Carolina Rayo, Yizhi Xiao, Walter L. Siqueira

**Affiliations:** 1 Schulich School of Medicine & Dentistry, University of Western Ontario, London, Ontario, Canada; 2 Department of Dental Clinic, Division of Graduate Periodontics, Federal University of Rio de Janeiro, Rio de Janeiro, Brazil; 3 Institute of Microbiology, Federal University of Rio de Janeiro, Rio de Janeiro, Brazil; University of North Carolina at Chapel Hill, United States of America

## Abstract

**Aim:**

To quantify the proteome composition of the GCF in periodontal health (HH) and in sites with different clinical conditions in chronic periodontitis (CP) subjects.

**Material and Methods:**

5 subjects with HH and 5 with CP were submitted to full-mouth periodontal examination, and GCF sampling. Sites in the CP group were classified and sampled as periodontitis (P, probing depth, PD>4 mm), gingivitis (G, PD≤3mm with bleeding on probing, BOP), and healthy sites (H, PD≤3mm without BOP). GCF proteins were subjected to liquid chromatography electrospray ionization mass spectrometry for identification, characterization and quantification.

**Results:**

230 proteins were identified; 145 proteins were detected in HH, 214 in P, 154 in G, and 133 in H. Four proteins were exclusively detected at HH, 43 proteins at P, 7 proteins at G, and 1 protein at H. Compared to HH group, 35 and 6 proteins were more abundant in P and G (p<0.001), respectively; and 4, 15 and 37 proteins were less abundant in P, G and H (p≤0.01), respectively.

**Conclusions:**

There are marked differences in the GCF proteome according to disease profile. Comprehension of the role of the identified proteins in the etiopathogenesis of periodontal disease may lead to biomarkers definition.

## Introduction

Clinical periodontal conditions are described as periodontal health (PH), gingivitis (G) and periodontitis (P) [1]. Gingivitis induced by dental biofilm is the presence of gingival inflammation without clinical attachment loss (CAL) and with no radiographic evidence of alveolar bone loss. Periodontitis is the presence of gingival inflammation with CAL, and re-absorption of the alveolar bone. In most patients, the increased probing depth (PD) or the formation of periodontal pockets follows the development of the periodontitis [1,2]. Chronic periodontitis (CP) is the most common destructive periodontal disease in adults [3].

The etiological agent of periodontitis is the high levels and proportions of periodontopathic bacteria in the subgingival biofilm [4,5]. The interplay between the pathogenic biofilm and the periodontal tissues in susceptible subjects leads to immunological responses that can be detected in the tissues and in the inflammatory exudates of the gingival crevicular fluid (GCF) [6,7]. In the GCF, pro-inflammatory cytokines and a vast array of other proteins can be found, especially in diseased subjects [8]. Recent reports have shown that, even in healthy periodontal microenvironment, GCF contains local proteins derived from extracellular matrix components and degradation products, as well as serum-derived proteins [7,9,10].

As GCF is an oral cavity-specific fluid, it has been studied in order to determine which constituents could be used as biomarkers for periodontal diagnosis or prognostic for the progression of periodontitis [6,8]. In the recent years, i t is been recognized that the multivariate model is more promising than a single biomarker for risk assessment of disease [11]. The ability to screen and discover multiple biomarkers simultaneously may provide a more valid clinical diagnosis and may be more useful for recognizing molecular patterns predictive for disease development. This multi-biomarker approach has progressed by recent advances in clinical proteomics [12–14]. Many studies have been performed in GCF analyzing its proteome profile through mass spectrometry (MS) technology [7,9,10,15–19]. Those studies differed in relation to the clinical condition, including studies of experimental gingivitis [15,16], aggressive periodontitis subjects [7], CP subjects [9,18,19], and healthy subjects [10]. Different findings are reported in those studies, not only because of differences inherent to periodontal condition but also because of the MS technique employed. On the other hand, to the moment, proteomics of GCF is in its beginning and there is a vast array of possible biomarkers [8,14]. Thus, comprehensive studies of GCF in different clinical conditions would contribute to a better understanding of the diagnostic potential of the GCF, improving the ability to early detection of disease [6,8,14].

Based on it, we hypothesized that the GCF proteome of PH subjects qualitatively and quantitatively differs from the proteome of CP subjects. This working hypothesis was explored by using quantitative proteomics approach based on label-free LC-MS on GCF of subjects with PH and CP; in CP subjects, sites with different clinical conditions as periodontal health, gingivitis and periodontitis were assessed.

## Materials and Methods

### Subject Population

Ten subjects who sought dental treatment in December of 2011 at the Dental School of the Federal University of Rio de Janeiro were enrolled in the present study. All participants were informed about the nature of the study and a signed consent form was obtained from each individual prior to entering into the study. The study protocol (#148/11) was reviewed and approved by the Review Committee for Human Subjects of the Clementino Fraga Filho University Hospital of the Federal University of Rio De Janeiro.

In order to participate of the study, subjects had to present at least 14 teeth, ≥ 18 years of age, and clinical diagnosis of CP or PH. Exclusion criteria included smoking, pregnancy, nursing, periodontal therapy in the last year, and use of antibiotics in the previous six months, as well as any immunological condition that could affect the progression of periodontitis. Individuals who required antibiotic coverage for routine periodontal procedures were also excluded. Furthermore, PH subjects should present clinical attachment level (CAL) characteristics as described bellow in order to avoid potential incipient destructive periodontal disease.

### Clinical evaluation

A calibrated examiner performed all clinical examinations. The intra-class correlation coefficient for CAL at the site was 0.90, and for probing depth (PD), 0.92. Full-mouth measurements including PD, CAL, presence or absence of supragingival biofilm (SB) and bleeding on probing (BOP) were recorded at six sites per tooth in all teeth, but third molars. Clinical diagnosis of periodontal status was established for all subjects based on the following criteria: periodontal health (PH), ≤ 10% of sites with BOP, no PD or CAL > 3 mm, although PD or CAL = 4 mm in up to 5% of the sites without BOP was allowed; and chronic periodontitis (CP), > 10% of teeth with PD and/or CAL ≥ 5 mm and BOP [20]. CP subjects had to have at least 5 sites with gingivitis (PD ≤ 3mm with BOP) and 4 sites with clinical periodontal health (PD ≤ 3mm without BOP).

### GCF sampling

Fourteen GCF samples from non-adjacent sites were collected from each participant. In CP subjects, different categories of sites were selected: 5 deep (PD >4 mm) sites (P), 5 shallow sites with BOP as gingivitis (G), and 4 shallow sites without BOP as health (H). In PH subjects, 14 buccal sites from the upper jaw were sampled (HH samples).

After removal of supragingival biofilm, the teeth were isolated with cotton rolls and a sterile Periopaper strip (ProFlow Inc., Amityville, NY, USA) was gently inserted into the selected subgingival sites and left there for 30s. Then, the volume of the GCF was measured using a Periotron 8000 (Oraflow Inc., Smithtown, NY, USA). The strips were stored in microcentrifuge tubes at -80°C.

### Sample preparation

For each CP subject, the paper strips were pooled according to the clinical categories (H, G and P) in microcentrifuge tubes. For PH subjects, two pools were made, dividing the 14 strips in two tubes. Each tube was incubated with 150 µL of a solution containing 80% acetonitrile, 19.9% distilled water and 0.1% trifluoroacetic acid and sonicated for 1 min. This procedure was repeated three times, in order to elute all proteins from the paper strips. Eluted proteins from sites of the same clinical category were pooled. In the end, there were three pools for the 5 CP subjects as P, G and H, and there was one pool for the 5 PH subjects. The pools were concentrated by a rotary evaporator. The total protein concentration was assessed by Micro Bicinchoninic acid (Micro BCA^TM^) Assay (Thermo Scientific, Rockford, USA). Equal protein amount (10 μg) from sites categories was dried by a rotary evaporator, denatured and reduced for 2 h by the addition of 200 µL of 4M urea, 10 mM dithiothreitol (DTT), 50 mM NH4HCO3, pH 7.8. After four-fold dilution with 50 mM NH4HCO3, pH 7.8, tryptic digestion was carried out for 18 h at 37°C, after the addition of 2% (w/w) sequencing-grade trypsin (Promega, Madison, WI, USA) [21].

### Liquid Chromatography Electrospray Ionization Tandem Mass Spectrometry (LC-ESI-MS/MS)

Equal amounts of all samples were dried by rotary evaporator and re-suspended in 20 µl of 97.5% H2O/2.4% acetonitrile/0.1% formic acid and then subjected to reversed-phase LC-ESI-MS/MS. Peptide separation and mass spectrometric analyses were carried out with a nano-HPLC Proxeon (Thermo Scientific, San Jose, CA, USA) which allows in-line LC with the capillary column, 75 µm x 10 cm (Pico Tip^TM^ EMITTER, New Objective, Woburn, MA, USA) packed in-house using Magic C18 resin of 5 µm diameter and 200 Å pores size (Michrom BioResources, Auburn, CA, USA) linked to mass spectrometer (LTQ Velos, Thermo Scientific, San Jose, CA, USA) using an electrospray ionization in a survey scan in the range of m/z values 390–2000 tandem MS/MS. The nano-flow reversed-phase HPLC was performed with linear 100 min gradient ranging from 5% to 55% of solvent B in 65 min (97.5% acetonitrile, 0.1% formic acid) at a flow rate of 300 nL/min with a maximum pressure of 280 bar. Electrospray voltage and the temperature of the ion transfer capillary were 1.8 kV and 250°C, respectively.

### Enzyme-linked immunosorbent assay (ELISA)

To validate the differential protein level identified by quantitative mass spectrometry approach, ELISA was carried out on lysozyme, a protein with well-known characteristics and functions in the oral cavity. ELISA microtiter plate (96-wells) was coated with 100 µl of GCF protein material from each group (10 µg/ml) at 37°C for 1 hour. The plate was then washed three times with 250 µl Tris Buffered Saline (TBS) per well and 200 µl TBST containing 3% BSA added to each well to block uncoated sites, and incubated overnight at 4°C. Primary anti lysozyme antibody (50 µl; 1:1000 dilution, Abcam, ab97950, MA, USA) in TBST containing 1% BSA was added to each well and incubated at 37°C for 1.5 h, followed by washing three times, and incubation with horse radish peroxidase (HRP) linked Anti- MOUSE IgG (H&L) (GOAT) Antibody Peroxidase Conjugated (100µl; 1:5000 dilution, ROCKLAND, PA, USA) in TBST containing 1% BSA. After incubation in the dark for 1 h at room temperature OPD (o-phenylenediamine dihydrochloride, Sigma-Aldrich, MO, USA) was added and product was analyzed spectrophotometrically at 490 nm. The experiment was performed in triplicate.

### Data analysis

A statistical program (SPSS Statistics 19, IBM Brazil, São Paulo, SP, Brazil) was used for clinical analysis. Full-mouth clinical data were averaged in each patient and within groups. Clinical parameters for the 14 sampled sites were also computed for each patient and averaged within groups. Significant differences in demographic and clinical parameters among groups were determined by Kruskal-Wallis, Mann-Whitney and χ^2^ tests. For MS data, each survey scan (MS) was followed by automated sequential selection of seven peptides for a standard collision-induced (CID) method, with dynamic exclusion of the previously selected ions. The obtained MS/MS spectra were searched against human protein databases (Swiss Prot and TrEMBL, Swiss Institute of Bioinformatics, Geneva, Switzerland, http://ca.expasy.org/sprot/) using SEQUEST algorithm in Proteome Discoverer 1.3 software (Thermo Scientific, San Jose, CA, USA), using at least two peptides. Search results were filtered for a False Discovery rate of 1% employing a decoy search strategy utilizing a reverse database [21]. The proteins identified were grouped into 9 different categories based on their known biological functions.

For quantitative proteome analysis, three MS raw files from each pooled clinical categories were analyzed using SIEVE software (Version 2.0 Thermo Scientific, San Jose, CA, USA). Signal processing was performed in a total of 12 MS raw files. The SIEVE experimental workflow was defined as ‘‘Control Compare Trend Analysis’’ where one class of samples are compared to one or more other classes of samples. In the present study, the HH group was compared to each of the other group (H, G and P). For the alignment step, a single MS raw file belonging to the HH group was selected as the reference file and all of the other files were adjusted to generate the best correlation to this reference file. After alignment, the feature detection and integration (or framing) process was performed using the MS level data with a feature called ‘‘Frames From MS2 Scans’’ only. When using this type of framing only MS mass-to-charge ratio (m/z) values that were associated with MS2 scan are used. Any m/z measurements that do not have MS2 were ignored. The parameters used consisted of a frame m/z width of 1500 ppm and a retention time width of 1.75 min. A total of 216,099 MS2 scans were present in all of the 12 RAW files that resulted in a total of 20,158 frames. Then peak integration was performed for each frame and these values were used for statistical analysis. Next, peptide sequences obtained from the database search using SEQUEST algorithm in Proteome Discoverer 1.3 were imported into SIEVE. A filter was applied to the peptide sequences during the import that eliminated all sequences with a Percolator q-value greater than 1% (false discovery rate). Peptides were grouped into proteins and a protein ratio and p-value were calculated, using a weighted average of the peptide intensities for the protein calculation. Only proteins observed in all four groups were quantified. HH group was used as the default group and all other three groups were compared with HH group. Relative abundance of an individual protein from HH group was considered significantly different protein level when the values observed were <0.75 for decreased abundance or >1.25 for increased abundance, and a p-value <0.05 as described previously [22,23].

For ELISA results, mean (± standard-deviation) values were calculated for each group. Afterwards, Analysis of Variance and Student-Newman-Keuls test for pairwise comparisons was carried out to identify significant differences among groups at a 5% level.

## Results

### Demographic and clinical findings

Table 1 shows the demographic and clinical data of the sample population. CP subjects presented significantly higher mean age than HH subjects (p < 0.01, Mann-Whitney test). Full-mouth clinical data show that CP had significantly higher mean PD and CAL (p < 0.01), and mean % of sites with BOP and SB than HH subjects. In fact, HH subjects presented no sites with BOP or SB. Regarding the clinical data of the sampled sites, significantly differences among sites from diseased subjects and HH subjects were detected (p < 0.01, Kruskal-Wallis test). Sites with periodontitis (P) presented the highest means for PD and CAL than the other categories; and all sites with gingivitis (G) presented BOP. The volume of GCF samples differed significantly among groups (p = 0.016). The GCF mean volume obtained from P sites of the CP group (0.3 µL ± 0.06) was significantly higher than samples from the HH group (0.1 µL ± 0.03, p = 0.016, Mann-Whitney test), H sites (0.06 µL ± 0.02, p = 0.009), and G sites (0.1 ± 0.04, p = 0.028). However, there was no significant difference between the HH group and G or H sites of CP.

**Table 1:** Demographic and clinical data (full-mouth and sampled sites; mean ± SEM) of the study population.

**Variables**	**Groups**			
	**CP**			**PH**
	(n = 5)			(n = 5)
Age (years)	46.20 ± 4.84			22.20 ± 0.66*
Gender - Females (%)	100			100
***Full mouth***				
PD (mm)	2.67 ± 0.13			1.16 ± 0.03*
CAL (mm)	2.78 ± 0.12			1.16 ± 0.03*
BOP (%)	51.51 ± 6.39			0*
Supragingival biofilm (%)	39.25 ± 7.24			0*
***Sampled sites***	**P**	**G**	**H**	**HH**
PD (mm)	5.16 ± 0.27	1.89 ± 0.19	1.62 ± 0.12	1.03 ± 0.05^†^
CAL (mm)	5.24 ± 0.25	2.01 ± 0.16	1.78 ± 0.19	1.03 ± 0.05^†^
BOP (%)	75.0 ± 14.66	100 ± 0.0	0	0^†^
Supragingival biofilm (%)	40.66 ± 12.93	41.57 ± 13.82	0	0^†^
GCF volume (μL)	0.3 ± 0.06^‡^	0.1 ± 0.04	0.06 ± 0.02	0.1 ± 0.03^§^

* p < 0.01, ^‡^p < 0.05, Mann-Whitney test; ^†^p < 0.01, ^§^p= 0.016, Kruskal-Wallis test; CP: chronic periodontitis; PH: periodontal health; P: periodontitis; G: gingivitis; H: health in periodontitis; HH: healthy sites in healthy subjects; PD: Probing depth; CAL: Clinical attachment level; Bleeding on probing; GCF: gingival crevicular fluid.

### Protein identification

**Figure 1:**
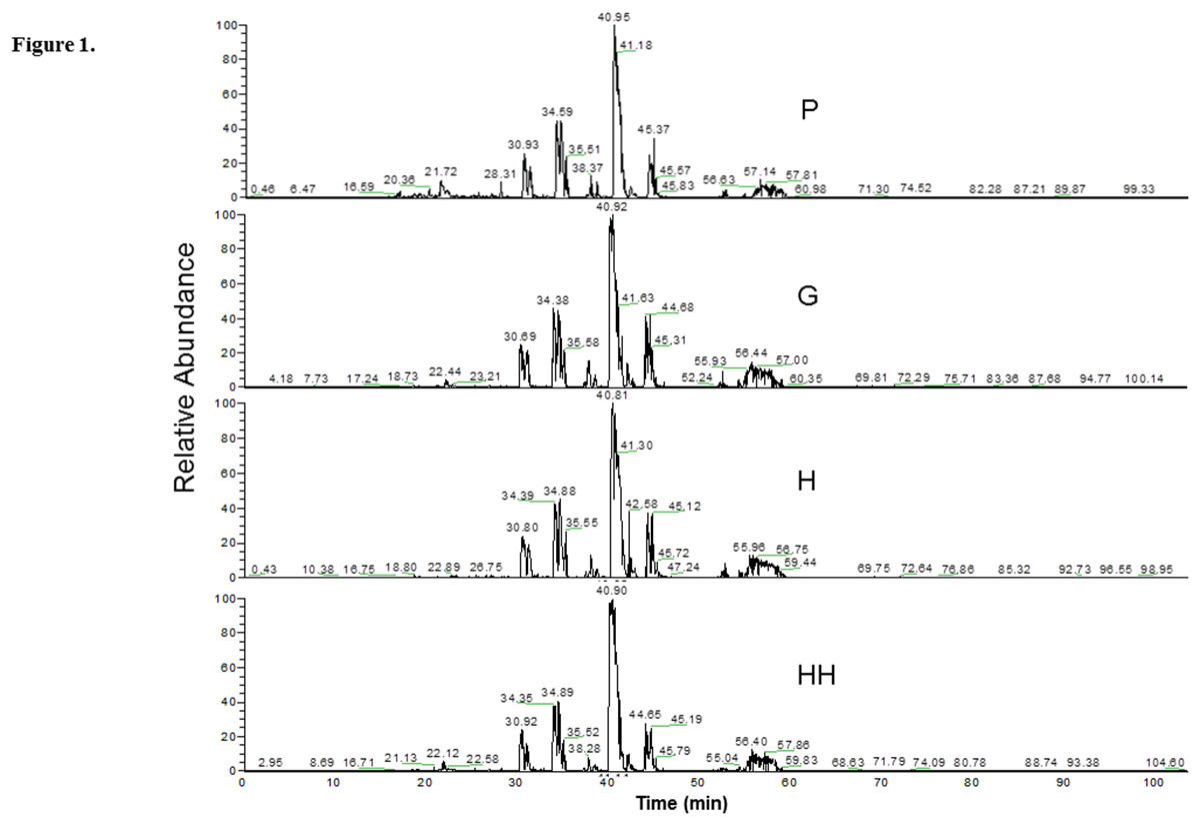
Examples of base-peak chromatograms of the clinical groups. Peptide separation was achieved using a nano-flow reverse-phase HPLC column, with gradient elution ranging from 5 to 55% solvent B in 100 min. P: sites with probing depth >4 mm; G: sites with probing depth ≤3 mm and bleeding on probing; H: sites with probing depth ≤3 mm without bleeding on probing in periodontitis subjects; HH: sites with probing depth ≤3 mm without bleeding on probing in healthy subjects.

After protein elution from the paper strip and trypsinization, equal amounts of peptides were subjected to nanoscale LC-ESI-MS/MS. A total of 3 runs per group were carried out. The base-peak chromatogram for reversed-phase chromatography monitored by the mass spectrometer represents the intensity of all peptide ions in the sample in a single scan. GCF proteome from all four different groups showed a consistent elution of protein/peptides range from 20 to 60 min (Figure 1).

**Figure 2:**
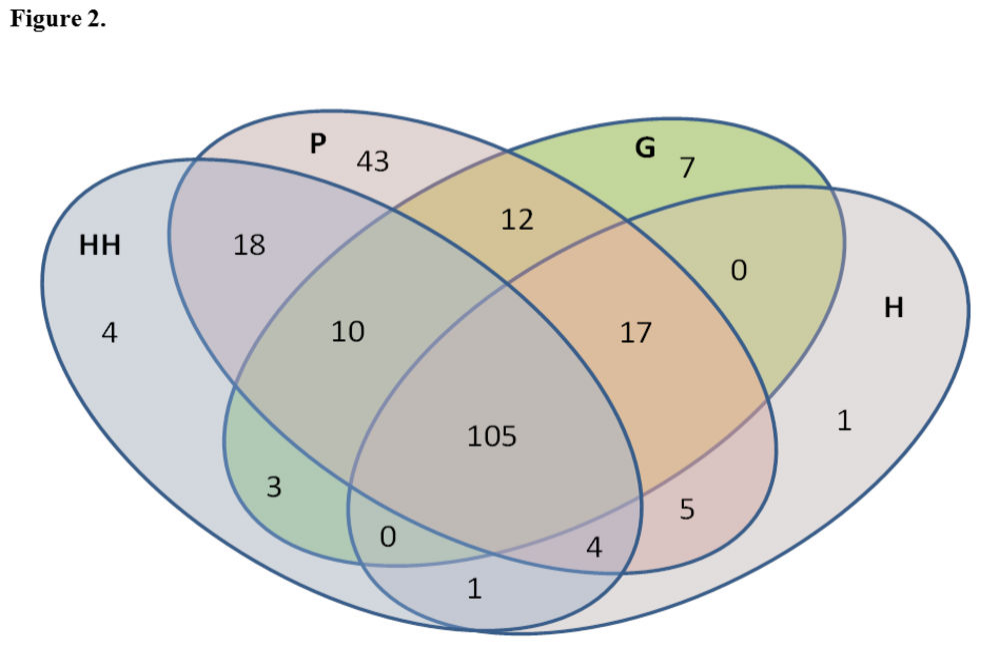
Venn diagram summarizing the absolute number of proteins detected in gingival crevicular fluid samples from periodontally healthy (145 proteins), and chronic periodontitis subjects in three categories P (deep probing depth sites, 214 proteins), G (shallow probing depth sites with bleeding on probing, 154 proteins) and H (shallow sites without bleeding on probing, 133 proteins).

A total of 230 different proteins were identified in GCF of all four groups (Table 2). Seventy proteins were related to cell differentiation, 60 to cell organization, 3 to coagulation, 1 to enzyme regulation, 36 to immune response, 23 to metabolism, 11 to signal transduction, 16 to transport, and 11 had unknown function. The HH group presented 145 proteins, while in the CP group P sites showed 214 proteins, G 154 proteins, and H 133 proteins. One hundred and five proteins were identified in all four groups, indicating a high overlap in GCF proteins (Figure 2). Four proteins were detected exclusively in the HH group, including keratin type II cytoskeletal 7, neuroblast differentiation-associated protein AHNAK, and 2 glial fibrillary acidic proteins. On the other hand, only one protein (nucleoprotein TPR) was detected in H sites. Moreover, 7 proteins were found exclusively in G sites: histones H1.1, H1.5 and H1t, fibrinogen alpha and beta chain, cathelicidin antimicrobial peptide, and myosin-9. Forty-three proteins were only detected in P sites and were distributed as follows: 10 were related to cell differentiation, 3 to cell organization, 1 to coagulation, 8 to immune response, 9 to metabolism, 8 to signal transduction, 2 to transport, and 2 had non-specified function (Figure 1 and Table 2).

**Table 2:** Gingival crevicular fluid proteome from periodontal health (HH, 145 proteins), chronic periodontitis in three categories P (deep probing depth sites, 214 proteins), G (shallow probing depth sites with bleeding on probing, 154 proteins) and H (shallow sites without bleeding on probing, 133 proteins), and number of hits of each protein achieved during three mass spectrometric runs.

Accession/ function	Protein name	Number of hits during three mass spectrometry runs			
		HH	P	G	H
*Cell differentiation*					
Q5T8M7	Actin, alpha 1, skeletal muscle	5	24	11	4
Q5T8M8	Actin, alpha 1, skeletal muscle	5	27	11	2
Q5T9N7	Actin, alpha 2, smooth muscle, aorta (fragment)	4	15	11	2
A6NL76	Actin, alpha skeletal muscle	5	27	11	7
E7EQV5	Actin, alpha skeletal muscle	11	22	11	2
P68133	Actin, alpha skeletal muscle	3	30	11	4
P62736	Actin, aortic smooth muscle	3	27	11	4
P60709	Actin, cytoplasmic 1	7	34	13	6
C9JTX5	Actin, cytoplasmic 1, N-terminally processed	12	12	11	2
C9JUM1	Actin, cytoplasmic 1, N-terminally processed	2	15	11	2
C9JZR7	Actin, cytoplasmic 1, N-terminally processed	12	15	11	2
E7EVS6	Actin, cytoplasmic 1, N-terminally processed	6	17	11	2
F5GYT4	Actin, cytoplasmic 1, N-terminally processed	0	22	0	0
P63261	Actin, cytoplasmic 2	7	34	13	6
F5H0N0	Actin, cytoplasmic 2, N-terminally processed	16	33	11	6
B8ZZJ2	Actin, gamma-enteric smooth muscle	4	12	11	2
C9JFL5	Actin, gamma-enteric smooth muscle	27	15	13	2
E9PG30	Actin, gamma-enteric smooth muscle	0	17	8	4
P63267	Actin, gamma-enteric smooth muscle	3	27	11	4
P13645	Keratin, type I cytoskeletal 10	61	46	41	62
C9JA77	Keratin, type I cytoskeletal 13	3	20	18	25
P13646	Keratin, type I cytoskeletal 13	44	23	25	39
P02533	Keratin, type I cytoskeletal 14	36	23	26	33
A8MT21	Keratin, type I cytoskeletal 15	2	5	0	8
B3KRA2	Keratin, type I cytoskeletal 15	8	5	0	13
P19012	Keratin, type I cytoskeletal 15	24	16	15	25
P08779	Keratin, type I cytoskeletal 16	30	14	16	21
Q04695	Keratin, type I cytoskeletal 17	12	8	6	14
C9JM50	Keratin, type I cytoskeletal 19	2	7	29	10
P08727	Keratin, type I cytoskeletal 19	16	13	13	16
P35900	Keratin, type I cytoskeletal 20	7	0	0	7
Q7Z3Z0	Keratin, type I cytoskeletal 25	4	4	7	2
Q7Z3Y8	Keratin, type I cytoskeletal 27	4	4	7	2
Q7Z3Y7	Keratin, type I cytoskeletal 28	4	4	7	2
P35527	Keratin, type I cytoskeletal 9	28	53	11	49
Q9NSB2	Keratin, type II cuticular Hb4	2	2	0	0
P04264	Keratin, type II cytoskeletal 1	86	71	40	88
Q7Z794	Keratin, type II cytoskeletal 1b	2	2	0	0
P35908	Keratin, type II cytoskeletal 2 epidermal	22	24	19	30
Q01546	Keratin, type II cytoskeletal 2 oral	4	5	7	7
P12035	Keratin, type II cytoskeletal 3	4	3	3	2
F5H8K9	Keratin, type II cytoskeletal 4	0	13	4	9
P19013	Keratin, type II cytoskeletal 4	8	13	4	9
E7EU87	Keratin, type II cytoskeletal 5	0	16	14	28
P13647	Keratin, type II cytoskeletal 5	25	20	17	30
E7EUE8	Keratin, type II cytoskeletal 6A	0	22	23	39
P02538	Keratin, type II cytoskeletal 6A	30	22	26	42
F5H6G5	Keratin, type II cytoskeletal 6B	0	13	14	34
P04259	Keratin, type II cytoskeletal 6B	30	18	19	40
E7EQV7	Keratin, type II cytoskeletal 6C	17	21	22	39
P48668	Keratin, type II cytoskeletal 6C	30	21	22	39
E7ES34	Keratin, type II cytoskeletal 7	0	8	0	0
F5GZD1	Keratin, type II cytoskeletal 7	2	0	0	0
P08729	Keratin, type II cytoskeletal 7	3	8	15	0
Q3SY84	Keratin, type II cytoskeletal 71	0	2	0	0
Q14CN4	Keratin, type II cytoskeletal 72	0	6	0	7
Q86Y46	Keratin, type II cytoskeletal 73	4	7	3	4
Q7RTS7	Keratin, type II cytoskeletal 74	0	2	0	2
O95678	Keratin, type II cytoskeletal 75	0	10	7	13
Q5XKE5	Keratin, type II cytoskeletal 79	5	7	7	8
P05787	Keratin, type II cytoskeletal 8	6	8	0	2
Q9H552	Keratin-8-like protein 1	0	2	0	0
Q09666	Neuroblast differentiation-associated protein AHNAK	2	0	0	0
Q0P5P4	TMSB4X protein (fragment)	2	2	2	0
Q5VU58	Tropomyosin 3	0	2	0	0
Q5VU66	Tropomyosin 3	0	2	0	0
Q5VU61	Tropomyosin 3	0	2	0	0
Q5VU72	Tropomyosin 3	0	2	0	0
P06753	Tropomyosin alpha-3 chain	0	2	0	0
P67936	Tropomyosin alpha-4 chain	0	2	0	0
*Cell organization*					
P02654	Apolipoprotein C-I	0	13	2	0
P02655	Apolipoprotein C-II	0	2	2	0
P02656	Apolipoprotein C-III	0	10	2	6
B0YIW2	Apolipoprotein C-III variant 1	2	10	8	2
P17661	Desmin	0	9	0	0
Q02539	Histone H1.1	0	0	2	0
P16403	Histone H1.2	10	8	11	2
P16402	Histone H1.3	10	8	11	2
P10412	Histone H1.4	10	8	11	2
P16401	Histone H1.5	0	0	2	0
P22492	Histone H1t	0	0	2	0
C9J386	Histone H2A	5	6	0	4
A6NFA8	Histone H2A	0	6	0	4
A6NKY0	Histone H2A	0	6	0	6
A6NN01	Histone H2A	11	10	3	8
C9J0D1	Histone H2A	4	10	3	10
C9JE22	Histone H2A	22	11	13	9
P0C0S8	Histone H2A type 1	12	20	17	15
Q96QV6	Histone H2A type 1-A	2	12	13	9
P04908	Histone H2A type 1-B/E	6	18	17	14
Q93077	Histone H2A type 1-C	6	18	17	14
P20671	Histone H2A type 1-D	12	20	19	15
Q96KK5	Histone H2A type 1-H	12	20	19	15
Q99878	Histone H2A type 1-J	12	20	19	15
Q6FI13	Histone H2A type 2-A	12	21	19	15
Q8IUE6	Histone H2A type 2-B	3	11	10	3
Q16777	Histone H2A type 2-C	12	21	19	15
Q7L7L0	Histone H2A type 3	6	18	17	14
Q9BTM1	Histone H2A.J	12	20	19	15
Q71UI9	Histone H2A.V	0	10	6	10
P16104	Histone H2A.x	2	12	13	9
P0C0S5	Histone H2A.Z	0	10	3	10
B4DR52	Histone H2B	4	17	6	4
Q96A08	Histone H2B type 1-A	2	3	0	0
P33778	Histone H2B type 1-B	7	14	8	4
P62807	Histone H2B type 1-C/E/F/G/I	11	17	8	4
P58876	Histone H2B type 1-D	11	17	8	4
Q93079	Histone H2B type 1-H	7	17	8	4
P06899	Histone H2B type 1-J	7	14	8	4
O60814	Histone H2B type 1-K	3	17	4	4
Q99880	Histone H2B type 1-L	7	17	8	4
Q99879	Histone H2B type 1-M	7	17	8	4
Q99877	Histone H2B type 1-N	7	17	8	4
P23527	Histone H2B type 1-O	19	14	20	23
Q16778	Histone H2B type 2-E	7	14	8	4
Q5QNW6	Histone H2B type 2-F	7	17	8	4
Q8N257	Histone H2B type 3-B	6	9	8	3
P57053	Histone H2B type F-S	11	17	8	4
B4DEB1	Histone H3	4	0	4	0
P68431	Histone H3.1	2	2	4	0
Q16695	Histone H3.1t	2	0	4	0
Q71DI3	Histone H3.2	2	4	4	0
P84243	Histone H3.3	2	0	4	0
P62805	Histone H4	26	30	29	23
P41219	Peripherin	0	4	0	0
P13796	Plastin-2	0	2	0	0
P07737	Profilin-1	0	4	2	0
P35542	Serum amyloid A-4 protein	0	2	11	0
P62328	Thymosin beta-4	2	2	2	0
A8MW06	Thymosin beta-4-like protein 3	6	2	2	0
*Coagulation*					
P02671	Fibrinogen alpha chain	0	0	3	0
P02675	Fibrinogen beta chain	0	0	2	0
Q9BYX7	Putative beta-actin-like protein 3	0	10	0	0
*Enzyme regulator*					
P04080	Cystatin-B	3	2	0	0
*Immune response*					
P01009	Alpha-1-antitrypsin	3	10	10	3
D6RA82	Annexin	2	3	0	0
D6RAZ8	Annexin	5	2	0	0
D6RCA8	Annexin	26	2	0	0
D6RFG5	Annexin	7	30	0	0
P04083	Annexin A1	17	39	18	10
Q5T3N0	Annexin A1 (fragment)	2	19	8	3
Q5T3N1	Annexin A1 (fragment)	10	28	13	9
P06727	Apolipoprotein A-IV	0	20	5	0
P49913	Cathelicidin antimicrobial peptide	0	0	2	0
P08311	Cathepsin G	7	19	17	4
P31146	Coronin-1A	2	6	0	0
P81605	Dermcidin	2	2	6	6
A5JHP3	Dermcidin isoform 2	2	2	6	26
B4DL87	Heat shock protein beta-1	32	7	0	0
C9J3N8	Heat shock protein beta-1	30	3	0	0
P04792	Heat shock protein beta-1	0	8	0	0
P01857	Ig gamma-1 chain C region	0	2	0	0
P0CG05	Ig lambda-2 chain C regions	0	3	12	0
P0CG06	Ig lambda-3 chain C regions	0	3	0	0
F5GWP8	Junction plakoglobin	0	7	9	14
B7Z4X2	Lactoferroxin-C	4	2	0	0
C9J0S5	Lactoferroxin-C	23	2	0	0
C9JCF5	Lactoferroxin-C	28	2	0	0
E7EQB2	Lactoferroxin-C	0	2	0	0
E7ER44	Lactoferroxin-C	0	2	0	0
E7ERT3	Lactoferroxin-C	0	2	0	0
P02788	Lactotransferrin	0	2	0	0
P30740	Leukocyte elastase inhibitor	0	3	0	0
P05164	Myeloperoxidase	8	31	2	0
P35579	Myosin-9	0	0	3	0
P59665	Neutrophil defensin 1	8	10	5	5
P59666	Neutrophil defensin 3	8	10	5	5
P12270	Nucleoprotein TPR	0	0	0	2
P05109	Protein S100-A8	5	17	7	2
P06702	Protein S100-A9	66	81	64	46
*Metabolism*					
P31946	14-3-3 protein beta/alpha	0	3	0	0
P31947	14-3-3 protein sigma	0	3	0	0
P27348	14-3-3 protein theta	0	3	0	0
F5H1C1	Actin, alpha cardiac muscle 1	11	24	14	4
P68032	Actin, alpha cardiac muscle 1	3	30	11	4
P02647	Apolipoprotein A-I	14	95	66	44
P02652	Apolipoprotein A-II	2	23	15	9
P02649	Apolipoprotein E	0	8	0	0
E9PK25	Cofilin-1	0	4	0	0
E9PLJ3	Cofilin-1	0	2	0	0
E9PP50	Cofilin-1	0	2	0	0
E9PQB7	Cofilin-1	0	2	0	0
P23528	Cofilin-1	0	4	0	0
P61626	Lysozyme C	3	13	7	2
P31949	Protein S100-A11	3	6	4	2
E7EW61	Transthyretin	0	20	11	0
F5H868	Transthyretin	0	19	5	0
P02766	Transthyretin	0	23	9	2
F5H288	Vimentin	0	37	12	0
P08670	Vimentin	9	37	10	0
Q5JVS8	Vimentin (fragment)	0	15	2	0
B0YJC4	Vimentin variant 3	7	37	10	0
B0YJC5	Vimentin variant 4	8	22	3	0
*Signal transduction*					
Q04917	14-3-3 protein eta	0	3	0	0
P61981	14-3-3 protein gamma	0	3	0	0
Q8IUK7	ALB protein	21	55	57	51
P12429	Annexin A3	0	5	0	0
E7EMB3	Calmodulin	0	2	0	0
E7ETZ0	Calmodulin	0	2	0	0
P62158	Calmodulin	0	2	0	0
P80511	Protein S100-A12	0	2	0	0
P31151	Protein S100-A7	0	2	0	0
E9PAX3	Glial fibrillary acidic protein	2	0	0	0
P14136	Glial fibrillary acidic protein	2	0	0	0
*Transport*					
C9JKR2	Albumin, isoform CRA-k	4	78	49	55
P69905	Hemoglobin subunit alpha	0	31	16	3
P68871	Hemoglobin subunit beta	0	50	24	20
E9PEW8	Hemoglobin subunit delta	0	23	22	0
E9PFT6	Hemoglobin subunit delta	0	23	8	0
P02042	Hemoglobin subunit delta	0	26	11	6
P25815	Protein S100-P	0	2	0	2
P02787	Serotransferrin	0	2	0	0
B7WNR0	Serum albumin	9	97	85	68
D6RHD5	Serum albumin	10	63	77	64
E7ESU5	Serum albumin	23	113	94	81
P02768	Serum albumin	28	113	94	81
D6RAK8	Vitamin D-binding protein	8	12	0	0
D6RF35	Vitamin D-binding protein	8	12	0	0
P02774	Vitamin D-binding protein	0	9	0	0
*Non specified*					
Q562R1	Beta-actin-like protein 2	0	10	0	0
B4DE78	cDNA FLJ52141, highly similar to 14-3-3 protein gamma	25	3	0	0
B4E335	cDNA FLJ52842, highly similar to Actin, cytoplasmic 1	21	33	13	6
B4DRW1	cDNA FLJ55805, highly similar to Keratin, type II cytoskeletal 4	4	13	8	9
Q6S8J3	POTE ankyrin domain family member E	0	15	7	2
A5A3E0	POTE ankyrin domain family member F	0	15	7	5
P0CG38	POTE ankyrin domain family member I	0	10	7	2
P0CG39	POTE ankyrin domain family member J	0	7	0	0
P02814	Submaxillary gland androgen-regulated protein 3B	4	4	0	0
A6NBZ8	Uncharacterized protein	24	106	90	54
A8MW45	Uncharacterized protein	11	7	9	4

The proteins identified were grouped into 9 different categories based on their known biological functions.

### Relative quantitative abundance ratios of proteins in GCF

The relative abundance ratios of the detected proteins in GCF samples from the studied groups are displayed in Table 3. Thirty-five proteins were found to be significantly high abundant in P sites of the CP compared to the HH group (p < 0.001). Many of them (12 proteins) were related to immune response, followed by metabolism (8 proteins), transport (6 proteins), cell differentiation (3 proteins), cell organization (3 proteins), signal transduction (2 proteins), and enzyme regulation (1 protein). Only 4 proteins were significantly low abundant in P sites compared to the HH group: 2 proteins with cell organization function (histone H2A, serum amyloid A 4 protein), and 2 proteins related to immune response (apolipoprotein AIV and myosin-9) (p < 0.001). Conversely, the G and H site categories had most proteins significantly low abundant compared to the HH group. In G sites, 37 proteins were significantly low abundant, mainly proteins related to cell differentiation (21 proteins), followed by immune response function (6 proteins) (p ≤ 0.01); and only 6 proteins were significantly high abundant (p ≤ 0.01). In H sites, 15 proteins were significantly low abundant (p ≤ 0.01), which included 5 proteins with cell organization function and 4 proteins with immune response function.

**Table 3:** Abundance* ratios of the detected proteins in gingival crevicular fluid samples from periodontally health (HH) and chronic periodontitis subjects (P: deep probing depth sites; G: shallow probing depth with bleeding on probing sites; and H: shallow probing depth without bleeding on probing sites).

**Accession/ function**	**Protein name**	**Ratio HH/HH**	**Ratio P/HH**	**p-value**	**Ratio G/HH**	**p-value**	**Ratio H/HH**	**p-value**
*Cell differenciation*								
E7EQV5	Actin, alpha skeletal muscle	1	2.19	< 0.001	-	-	-	-
F5GYT4	Actin, cytoplasmic 1, N-terminally processed	1	3.24	< 0.001	-	-	-	-
E9PG30	Actin, gamma-enteric smooth muscle	1	2.09	< 0.001	0.53	< 0.001	0.35	< 0.001
P35527	Keratin, type I cytoskeletal 9	1	-	-	0.71	< 0.001	-	-
P13645	Keratin, type I cytoskeletal 10	1	-	-	0.65	< 0.001	-	-
C9JA77	Keratin, type I cytoskeletal 13	1	-	-	0.66	< 0.001	-	-
P13646	Keratin, type I cytoskeletal 13	1	-	-	0.38	< 0.001	-	-
P02533	Keratin, type I cytoskeletal 14	1	-	-	0.66	< 0.001	-	-
A8MT21	Keratin, type I cytoskeletal 15	1	-	-	-	-	0.48	< 0.001
P08779	Keratin, type I cytoskeletal 16	1	-	-	0.57	< 0.001	-	-
C9JM50	Keratin, type I cytoskeletal 19	1	-	-	0.56	< 0.001	-	-
Q7Z3Z0	Keratin, type I cytoskeletal 25	1	-	-	0.47	< 0.001	-	-
Q7Z3Y7	Keratin, type I cytoskeletal 28	1	-	-	0.55	< 0.001	-	-
P04264	Keratin, type II cytoskeletal 1	1	-	-	0.69	< 0.001	-	-
P35908	Keratin, type II cytoskeletal 2 epidermal	1	-	-	0.73	< 0.001	-	-
Q01546	Keratin, type II cytoskeletal 2 oral	1	-	-	0.56	0.008	-	-
P19013	Keratin, type II cytoskeletal 4	1	-	-	0.52	0.009	-	-
P13647	Keratin, type II cytoskeletal 5	1	-	-	0.62	< 0.001	-	-
E7EU87	Keratin, type II cytoskeletal 5	1	-	-	0.65	< 0.001	-	-
E7EUE8	Keratin, type II cytoskeletal 6A	1	-	-	0.50	< 0.001	-	-
P02538	Keratin, type II cytoskeletal 6A	1	-	-	0.60	< 0.001	-	-
F5H6G5	Keratin, type II cytoskeletal 6B	1	-	-	0.51	< 0.001	-	-
E7EQV7	Keratin, type II cytoskeletal 6C	1	-	-	0.56	< 0.001	-	-
E7ES34	Keratin, type II cytoskeletal 7	1	-	-	0.52	< 0.001	-	-
*Cell organization*								
B0YIW2	Apolipoprotein CIII variant 1	1					0.74	< 0.001
P10412	Histone H1.4	1	2.11	< 0.001	0.64	< 0.001	0.60	< 0.001
P16401	Histone H1.5	1	-	-	1.56	< 0.001	-	-
P22492	Histone H1t	1	-	-	0.43	0.016	-	-
C9JE22	Histone H2A	1	0.70	< 0.001	0.68	< 0.001	0.44	< 0.001
Q9BTM1	Histone H2A.J	1	-	-	1.67	< 0.001	-	-
P16104	Histone H2A.x	1	-	-	-	-	-	-
B4DR52	Histone H2B	1	1.84	< 0.001	-	-	0.74	0.001
B4DEB1	Histone H3	1	-	-	0.47	< 0.001	-	-
P62805	Histone H4	1	-	-	-	-	0.42	< 0.001
P07737	Profilin 1	1	2.83	< 0.001	-	-	-	-
P35542	Serum amyloid A 4 protein	1	0.11	< 0.001	-	-	-	-
*Enzyme regulator*								
P04080	Cystatin B	1	1.78	< 0.001	0.61	< 0.001	0.27	< 0.001
*Immune response*								
P01009	Alpha 1 antitrypsin	1	2.16	< 0.001			-	-
D6RCA8	Annexin	1	1.59	< 0.001	-	-	-	-
P04083	Annexin A1	1	0.74	< 0.001	0.70	< 0.001	0.69	0.003
P06727	Apolipoprotein AIV	1	1.56	< 0.001	0.51	< 0.001	-	-
P49913	Cathelicidin antimicrobial peptide	1	1.44	< 0.001	-	-	-	-
P08311	Cathepsin G	1	2.27	< 0.001	-	-	-	-
P31146	Coronin-1A	1	3.06	< 0.001	-	-	0.66	< 0.001
A5JHP3	Dermcidin isoform 2	1	-	-	0.5	< 0.001	0.42	< 0.001
B4DL87	Heat shock protein beta-1	1	1.56	< 0.001	-	-	-	-
C9J3N8	Heat shock protein beta-1	1	1.80	< 0.001	-	-	-	-
P05164	Myeloperoxidase	1	1.80	< 0.001	-	-	-	-
P35579	Myosin 9	1	0.73	< 0.001	0.62	< 0.001	-	-
P59666	Neutrophil defensin 3	1	1.77	< 0.001	0.48	< 0.001	0.44	< 0.001
P05109	Protein S100 A8	1	1.26	< 0.001	-	-	-	-
P06702	Protein S100 A9	1	1.46	< 0.001	0.57	< 0.001	-	-
*Metabolism*								
P02647	Apolipoprotein AI	1	1.36	< 0.001	-	-	0.33	< 0.001
P02652	Apolipoprotein AII	1	3.05	< 0.001	-	-	-	-
P02649	Apolipoprotein E	1	2.97	< 0.001	-	-	-	-
E9PLJ3	Cofilin-1	1	2.81	< 0.001	-	-	-	-
P61626	Lysozyme C	1	2.13	< 0.001	0.71	< 0.001	0.29	< 0.001
P31949	Protein S100 A11	1	2.77	< 0.001	0.51	< 0.001	-	-
F5H868	Transthyretin	1	1.65	< 0.001	-	-	-	-
B0YJC4	Vimentin variant 3	1	2.20	< 0.001	-	-	-	-
*Signal transduction*								
E7ETZ0	Calmodulin	1	1.67	0.001	-	-	-	-
E9PAX3	Glial fibrillary acidic protein	1	-	-	1.47	< 0.001	-	-
P80511	Protein S100 A12	1	1.99	0.002	0.50	0.020	-	-
*Transport*								
P69905	Hemoglobin subunit alpha	1	2.35	< 0.001	1.43	< 0.001	-	-
E9PFT6	Hemoglobin subunit delta	1	1.95	< 0.001	-	-	-	-
P02042	Hemoglobin subunit delta	1	-		1.33	0.005	-	-
P25815	Protein S100 P	1	2.07	< 0.001	0.69	< 0.001	-	-
P02787	Serotransferrin	1	1.80	< 0.001	-	-	-	-
B7WNR0	Serum albumin	1	2.74	< 0.001	-	-	0.68	< 0.001
D6RF35	Vitamin D-binding protein	1	2.06	< 0.001	0.71	< 0.001	-	-
*Non specified*								
A6NBZ8	Uncharacterized protein	1	-	-	1.52	< 0.001	-	-

* Relative abundance of an individual protein from HH group was considered significant protein level when the values observed were < 0.75 for decreased abundance or > 1.25 for increased abundance, and a p-value < 0.05. The proteins identified were grouped into 9 different categories based on their known biological functions.

### ELISA

**Figure 3:**
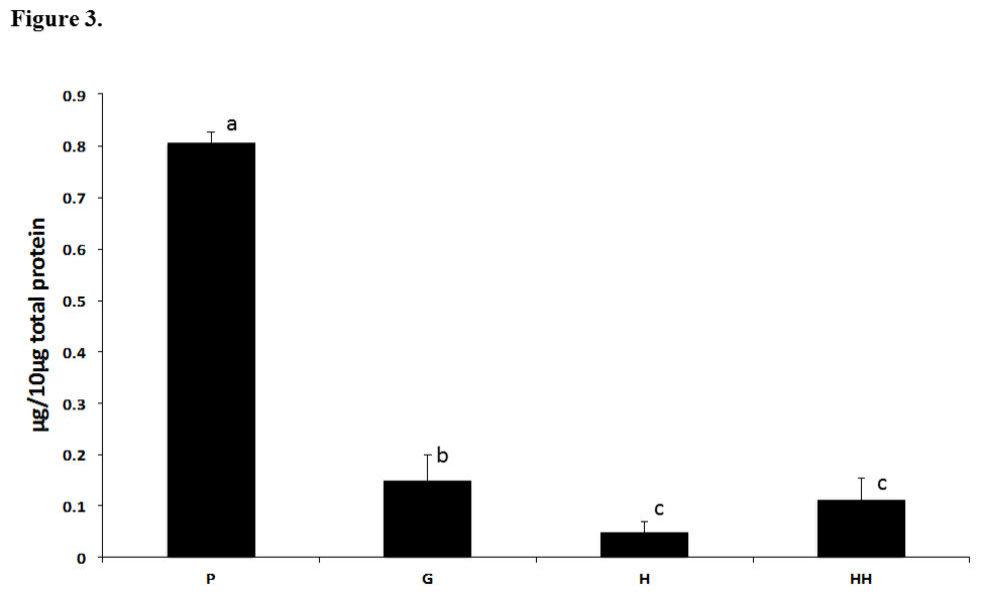
ELISA experiment with 10 μg of GCF material for each group and anti-lysozyme antibody. Bars represent standard deviation of the mean, calculated from three independent experiments. Different lower case letters denote statistical difference according to Analysis of variance and Student-Newman-Keels’ test. P: sites with probing depth >4 mm; G: sites with probing depth ≤3 mm and bleeding on probing; H: sites with probing depth ≤3 mm without bleeding on probing in periodontitis subjects; HH: sites with probing depth ≤3 mm without bleeding on probing in healthy subjects.

A significant reduced protein level was observed by MS at H and HH concentration compared to control G and P groups (Table 3). By ELISA Lysozyme contents were demonstrated to decrease from 0.805 ± 0.021 μg/10 μg GCF total protein in the P group, to 0.15 ± 0.05, 0.05 ± 0.02 and 0.113 ± 0.04 in the G, H, HH groups, respectively (Figure 3).

## Discussion

The aim of the present case-control study was to explore the human proteins present in GCF from healthy subjects as well as in subjects with CP. Additionally, analysis of GCF in different clinical conditions in CP subjects was possible because of the sampling strategy. Although, case-control studies may present limited level of evidence, they are often used to identify factors that may contribute to the disease development. The design experiment and the outcome data belong to the primary phase of five consecutive phases. Phase one, as preclinical exploratory studies, is the first (initial) step for the search of biomarkers [24].

The current study identified 230 proteins. In previous study [19], a similar amount of 231 proteins was identified; 168 proteins from those were identified in GCF samples from a healthy site, and 167 proteins were detected in periodontitis sites. Sixty-four proteins were contained only in the healthy samples, and 63 proteins were detected only in the periodontitis samples. In the current analysis, 4 proteins were detected exclusively in the HH group, and 43 proteins were only detected in P sites. The different amount of proteins detected in healthy subjects between studies could be explained by the number of subjects sampled, where in the previous study only one healthy subject was analyzed [19]. It has been demonstrated that when GCF is pooled from multiple sites, the site-specific variability in GCF constituents is reduced [10,17]. Perhaps, by increasing the number of subjects sampled, it is possible that the range of proteins detected increases. Other studies that evaluate the GCF from healthy or diseased subjects have reported a lower number of detected proteins [7,10,15–17], while others have identified a higher amount [9,18]. In the study of Baliban et al. [9], 432 human proteins were found. From those, 79 were exclusively found in healthy subjects and 123 in periodontitis subjects. Another study have identified 327 human proteins in healthy subjects [18]. The difference in the amount of identified proteins can also be explained by the MS technique employed as well as the number of peptides used to identify the proteins. Studies that have used only one peptide to identify proteins may have increased the sensibility [9,18], however it may have reduced the specificity.

The most frequently detected proteins in the current study were actins, keratins, histones, annexins, protein S100-A9, apolipoprotein A-I, ALB protein, albumin and serum albumin. These findings are in accordance with a prior proteome study [9], in which the proteins more often detected in both healthy and CP samples were serum albumin, serotransferrin and α-2-macroglobulin. In that study, the authors have also found a wide variety of Type I and II keratins. The high frequency of these proteins observed in the current and other investigations may reflect the high turnover and rate of differentiation of oral epithelia [7,9]. In contrast, cytokines were not detected, as reported also by previous studies applying proteomic based mass spectrometric [7,9,16]. Possible explanations for this finding may be their low concentrations or low molecular weight, for which a drop of peptide signal is still a limiting factor for detection or being masked by the presence of other proteins as albumin [7,9,16,25].

An efficient periodontal biomarker should be able to predict future attachment loss in a susceptible subject. Therefore, the analysis of gingivitis sites in at risk subjects might help in the identification of which factors are already present and what might indicate future breakdown. Having that in mind, some proteins only found in G sites of CP subjects when two peptides were used should be highlighted as histones H1.1, H1.5 and H1t, fibrinogen alpha and beta chain, cathelicidin antimicrobial peptide, and myosin-9. Additionally, some other proteins that were relative more abundant in G compared to the HH group such as histone H2A.J, and glial fibrillary acidic protein might also be important (p <0.005). Proteins of the intermediate filament family as glial fibrillary acidic protein, peripherin, and desmin bind together to form intermediate filaments, providing support and strength to cells, and determining the placement of the nucleus and other specialized structures within the cell. Kido et al. [19] have described high levels of actin and myosin related proteins in GCF samples mainly from periodontitis subjects. Those proteins may be related to periodontal tissue degradation and inflammation [19]. Furthermore, myosin-9 can stimulate leukocyte migration and monocyte differentiation [10]. Myosin-9 and fibrinogen alpha chain were detected only in the resolution phase of experimental gingivitis, while histone H1.5 was detected in relative high frequency in both phases (induction and resolution) [15]. In another experimental gingivitis study, the authors also found myosin-9, fibrinogen alpha and beta chains, histone H1.5 and cathelicidin antimicrobial peptide [16]. Among those proteins, only cathelicidin antimicrobial peptide has been described as involved in periodontal disease [26]. Most recently, histones have been shown to play an important role in an extracellular defense mechanism, neutrophil extracellular traps (NETs) [27]. Moreover, in 2010 it was the first time that a histone protein was reported in GCF [17]. Low concentrations of histone H2A-histone H2B-DNA complexes may present antibacterial property against the species *Shigella flexneri, Salmonella typhimurium*, and *Staphylococcus aureus* [27]. Moreover, NETs contain other proteins such as neutrophil elastase, cathepsin G, myeloperoxidase, lactoferrin, and gelatinase [27]. Additionally, the outflow of GCF associate to NETs may be an important mechanism of clearance of the gingival sulcus or periodontal pocket [28].

From the 214 proteins detected in P sites, 43 were exclusively detected in this category. It is known that greater pocket depths associated to clinical signs of inflammation (i.e. bleeding) may result in increased levels of protein-breakdown products expressed in GCF [17]. Those 43 proteins detected included actin cytoplasmatic, tropomyosins, plastin-2, putative beta-actin-like protein 3, peripherin, desmin, Ig gamma-1 and -3 chain C regions, lactoferroxin-C, lactrotransferrin, leukocyte elastase inhibitor, 14-3-3 protein beta/ alpha, 14-3-3 protein sigma, 14-3-3 protein theta, 14-3-3 protein eta, 14-3-3 protein gamma, apolipoprotein E, cofilin-1, calmodulin, annexin A3, protein S100-A12, protein S100-A7, serotransferrin, and vitamin D-binding protein. In another analysis, many proteins showed significant higher relative abundance in P sites when compared to the HH group as alpha 1 antitrypsin, annexin, apolipoprotein AIV, cathelicidin antimicrobial peptide, cathepsin G, coronin-1A, dermcidin isoform 2, heat shock protein beta-1, profilin 1, myeloperoxidase, neutrophil defensin 3, S100 A8, S100 A9, S100 P, vitamin D-binding, serotransferrin (p < 0.001). Two histones (H1.4 and H2B) had also higher relative abundance in P sites than sites from HH patients. Actin and actin binding proteins as profilin and cofilin were described for the first time in GCF samples, which may be representing an osteoblastic bone activity [17]. In addition, other GCF proteins were also identified previously and confirmed in the current investigation as alpha 1 antitrypsin, cofilin-1, profilin 1, cathelicidin antimicrobial peptide, and heat shock protein in chronic periodontitis subjects [17]. In another study, cathepsin G, cathelicidin antimicrobial peptide, protein S100-A7, 14-3-3 protein sigma and vitamin D-binding were found more frequently in chronic periodontitis when compared to healthy subjects [9]. All those proteins were identified and characterized in the current investigation. Moreover, dermcidin was identified only in chronic periodontitis subjects.

As it should be expected, proteins related to immune response as Ig gamma-1 chain C region, Ig gamma-3 chain C region, lactoferroxin-C, lactrotransferrin, leukocyte elastase inhibitor, apolipoprotein E, alpha 1 antitrypsin, annexin, cathelicidin antimicrobial peptide, cathepsin G, coronin-1A, dermcidin isoform 2, heat shock protein beta-1, myeloperoxidase, neutrophil defensin 3, S100 A8, and S100 A9 were present in samples from deep pockets and/or have elevated relative abundance compared to samples from healthy sites (Table 3). For instance, dermcidin isoform 2 displays antimicrobial activity and is highly effective against *Escherichia coli, Enterococcus faecalis*, *S.* aureus and *Candida albicans* [29]. Surprisingly, two proteins of the immune system, Annexin A1 and myosin 9, showed significantly decreased relative abundance in P sites compared to HH group (Table 3, p < 0.001). Conversely, other studies have shown that myosin 9 was found only in disease [7] or in higher frequency in GCF of periodontitis subjects [9]. In a study of periodontally healthy and generalized aggressive periodontitis subjects, a total of 101 human proteins was found in GCF, 35 from those were exclusively detected in aggressive periodontitis [7]. In accordance with the current findings, the authors found that annexin A3, cathepsin G, and S100 P were identified only in diseased subjects, and that myeloperoxidase and profilin 1 were up-regulated in disease. In contrast, the proteins serotransferrin and alpha 1 antitrypsin were low abundant in disease, while neutrophil defensin 3 was detected just in healthy subjects. However, comparisons between those studies should be interpreted carefully, given that aggressive periodontitis subjects may present a more severe condition which is modulated by a genetic pre-disposition and different factors compared to chronic periodontitis subjects [1,2,30].

Findings from the HH group showed that a total of 145 proteins were detected, and only 4 proteins were exclusively found in that group. In the analysis of abundance ratios, it was interesting to observe that all significant results showed that the detected proteins in H sites were low abundant compared to the HH group. Although, the sites sampled were clinically similar in both groups, with no attachment loss or BOP, metabolically they are completely different by the expression of the proteins actin, apolipoprotein, histones, cystatin B, annexin, coronin-1A, dermcidin isoform 2, neutrophil defensin 3, and lysozyme C. Many of those proteins are related to the immune system. Although there is a clinical similarity, in a susceptible diseased subject healthy sites may not be very effective in the immune response as in a healthy subject. Other authors have also found a great number of proteins in GCF from healthy subjects [7,9,10,18], where cofilin 1, profilin 1, plastin 1, lactotransferrin, myeloperoxidase, calmodulin and alpha 1 antitrypsin were more frequently detected in samples from health subjects compared to CP [9]. Surprisingly, they found leukocyte elastase inhibitor only in healthy subjects. In the current study, one type of heat shock protein beta-1 was detected in P sites, and higher frequency of detection of other two heat shock protein beta-1 was detected in HH patients. Likewise, other authors have identified heat shock protein beta-1 in healthy subjects [16]. Conversely, Bostanci et al. [7] detected heat chock protein only in aggressive periodontitis subjects. The proteins S100A8 and S100A9, which are produced by neutrophils and macrophages, can be found in plasma, but their levels in GCF may differ significantly during periodontal disease owing to the local inflammatory response and recruitment of those white cells [10]. This may reinforce the idea that even though serum contributes to its composition, the GCF from healthy periodontal sites is neither pure serum nor are its proteins all serum derived [10].

Although our sample size is not large, it was enough to show significant differences among the clinical groups through MS analysis. Moreover, the differential protein levels identified among groups were confirmed by ELISA on the levels of Lysozyme. On the other hand, the studies previously cited in the current report were performed in relatively small sample size as well. For instance, Bostanci et al. [7] have studied 5 healthy subjects and 5 aggressive periodontitis subjects; and Kido et al. [19] have studied 8 subjects with CP and 1 with periodontal health. Additionally, some studies have analyzed only 10 healthy subjects [10] or 12 CP subjects under maintenance therapy [17].

In general, an increase in gingival inflammation results in an increase in GCF flow, and differences exist between GCF obtained from stable and progressing sites [17]. The current results demonstrated that there are markedly differences in the human proteome of GCF according to disease profile. Therefore, more studies completing the phases described by Pepe et al. [24], including multicenter studies [31], are necessary to understand the role of this vast range of identified proteins in the etiopathogenesis of periodontal disease. Through that comprehension, the discrimination of biomarkers for diagnosis and prognosis of periodontal diseases might be possible.
